# Partner presence, social support and chronic pain recovery: reply to a Letter to the Editor

**DOI:** 10.1016/j.bjane.2026.844819

**Published:** 2026-08-14

**Authors:** Camila C. Castro, Sandro Max C. Silva, Martha M.C. Castro, Durval Campos Kraychete, Carla Daltro

**Affiliations:** a Universidade Federal da Bahia, Medicine and Public Health, Salvador, BA, Brazil; b Universidade Federal da Bahia, Graduate Program in Integrative Processes of Organs and Systems, Salvador, BA, Brazil; c Universidade Federal da Bahia, Physiotherapy Course, Salvador, BA, Brazil; d Universidade Federal da Bahia, Graduate Program in Medicine and Health, Salvador, BA, Brazil

*Dear Editor*,

We sincerely thank the authors for their thoughtful comments on our study and for the opportunity to further discuss the influence of interpersonal relationships on the clinical course of patients with chronic pain.^1^ The letter raises important considerations regarding the interpretation of the association between partner presence and clinically meaningful pain improvement observed in our cohort.^2^

Partner presence should not be interpreted as a direct measure of social support. In our study, marital status was collected as a sociodemographic variable and included in the multivariable model because of its potential association with clinical outcomes. However, this study did not assess relationship quality, emotional or financial support, or the broader social support network of participants. Therefore, our findings do not allow us to infer that the observed association reflects a specific partner effect, nor do they permit the identification of the mechanisms through which partner presence may influence recovery.

Indeed, the literature demonstrates that the influence of interpersonal relationships on chronic pain is more complex than the simple presence or absence of a partner. A recent systematic review showed that the type of support provided by a partner may help explain differences in pain-related outcomes.^3^ Overly protective or pain-reinforcing responses may contribute to disability and symptom maintenance, whereas behaviors that encourage autonomy, adaptive coping strategies, and continued engagement in daily activities are associated with better functional outcomes.

Building on this line of research, we are currently conducting a prospective cohort study at the same center, where perceived social support and family climate are being assessed using validated instruments, as well as standard clinical outcomes. Although the results of this study are currently not available, we expect it to shed light on how different aspects of interpersonal relationships influence response to chronic pain treatment and whether the association identified in our previous study is driven by particular characteristics of social support and the family environment and not by the simple presence of a partner.

In our sample, no statistically significant difference was observed in SF-36 Social Functioning scores between patients with and without a partner (median [IQR], 37.25 [15.25–50.00] vs. 42.50 [25.00–63.00]; p = 0.397). This finding may reflect the broad scope of the SF-36 Social Functioning domain, which assesses the extent to which health interferes with social activities in a broad sense, encompassing family, occupational, and community interactions rather than exclusively intimate relationships. Thus, the observed improvement may reflect social reintegration resulting from pain reduction and overall improvement in physical and psychological functioning, rather than a specific effect of living with a partner.

We also appreciate the comment regarding medication adherence. As discussed in the original manuscript, adherence was not systematically assessed and therefore could not be included in our analyses. It is plausible that the presence of a partner or caregiver facilitates the management of complex therapeutic regimens, attendance at medical appointments, and adherence to non-pharmacological interventions. However, since these variables were not measured, any hypothesis that treatment adherence mediates this association remains speculative. We consider this an important issue for future prospective research.

Thus, we believe that our findings should be interpreted not as evidence of a causal effect of partner presence, but rather as an indication that interpersonal context may represent a factor associated with the clinical course of chronic pain. It is widely recognized as a condition influenced by biological, psychological, and social determinants that interact dynamically over time. In this context, identifying marital status as an independent predictor of clinical improvement does not imply that the relationship itself is the causal mechanism; rather, it suggests that this variable may serve as a proxy for broader interpersonal and social dimensions that warrant further investigation through more comprehensive psychosocial assessments.

We thank the authors once again for their valuable observations, which enrich the interpretation of our findings and reinforce the importance of further investigation into the interpersonal and social determinants of recovery in patients with chronic pain.

## Data availability statement

No new dataset was generated for this Letter. The additional aggregate analysis reported here was derived from the dataset of the original study and is available from the corresponding author upon reasonable request, subject to the same ethical restrictions described in the original article.

## AI assistance disclosure

During the preparation of this work, the authors used OpenAI exclusively for language editing and to improve the clarity of the manuscript. After using this tool, the authors reviewed and edited the content as necessary and assume full responsibility for its content.

## Conflicts of interest

The authors declare no conflicts of interest.
